# Contamination rates in serially sampled sputum specimens obtained during tuberculosis treatment to capture culture conversion

**DOI:** 10.1128/spectrum.00969-25

**Published:** 2025-08-29

**Authors:** N. Niemand, J. A. Rooney, S. Malatesta, N. Rawoot, T. C. Bouton, E. J. Ragan, T. Carney, L. F. White, M. Farhat, C. R. Horsburgh, B. Myers, R. M. Warren, K. R. Jacobson

**Affiliations:** 1 South African Medical Research Council Centre for Tuberculosis Research, Division of Molecular Biology and Human Genetics, Faculty of Medicine and Health Sciences, Stellenbosch University, Cape Town, South Africa; 2 Department of Environmental Health, Harvard T H Chan School of Public Health 218854, Boston, Massachusetts, USA; 3 Department of Biostatistics, Boston University School of Public Health 27118, Boston, Massachusetts, USA; 4 Department of Medicine, Section of Infectious Diseases, Boston University Chobanian & Avedisian School of Medicine 12259, Boston, Massachusetts, USA; 5 Mental Health, Alcohol, Substance Use and Tobacco Research Unit, South African Medical Research Council 648869 , Tygerberg, South Africa; 6 Department of Psychiatry and Mental Health, University of Cape Town, Groote Schuur Hospital, Observatory, Cape Town, South Africa; 7 Department of Psychology, University of Johannesburg 61799, Johannesburg, South Africa; 8 Department of Biomedical Informatics, Harvard Medical School 1811 , Boston, Massachusetts, USA; 9 Department of Epidemiology, Biostatistics and Global Health, Boston University School of Public Health 27118, Boston, Massachusetts, USA; 10 Curtin enAble Institute, Faculty of Health Sciences, Curtin University 1649, Perth, Western Australia, Australia; 11 West Australian Country Health Service (WACHS)-Curtin University Research and Innovation Alliance, Curtin University 1649, Perth, Western Australia, Australia; Duke University, Durham, North Carolina, USA

**Keywords:** tuberculosis, contamination rates, sputum, diagnosis

## Abstract

Sputum cultures are the gold standard for tuberculosis (TB) diagnosis and treatment monitoring. However, cultures in MGIT liquid media are susceptible to microbial contamination, often rendering them uninterpretable. Research has shown that maintaining strict cold chains and supervised sample collection can reduce contamination rates, but few longitudinal studies with weekly sampling have explored this. Here, we evaluated whether (i) the time between specimen collection and laboratory processing and (ii) unsupervised specimen collection are associated with contamination rates. Additionally, we estimated contamination rates over the first 12 weeks of treatment and assessed the clinical and behavioral predictors of contamination. We collected 3,155 sputum specimens from 301 participants undergoing TB treatment. Contamination was lowest (12.3%) at treatment initiation, increased over the first few weeks, and stabilized around 30% from week 8 onwards. Samples collected without supervision were more likely to be contaminated at treatment initiation (*P* = 0.048) and over 12 weeks (*P* = 0.028). We observed an inverse relationship between smear grade and contamination risk throughout the sampling period. These findings underscore the importance of supervised sputum collection to reduce contamination and provide ways to enhance the clinical and research values of weekly cultures, particularly those collected later in treatment. This is especially relevant for community-collected specimens used in monitoring treatment response.

**IMPORTANCE:**

It is essential to understand how we can minimize sputum specimen contamination rates, as culture contamination may lead to false negative or indeterminate results that require repeat sampling and testing, increasing the burden on healthcare systems and potentially delaying treatment initiation. This research underscores the importance of maintaining a stringent cold chain and highlights the need for participant education and supervision during sample collection. The findings from this study have important implications for TB diagnosis and treatment outcome programs in low- and middle-income countries where biosafety level 3 facilities are located within centralized national reference laboratories or tertiary care hospitals.

## INTRODUCTION

Sputum culture remains the preferred method for *Mycobacterium tuberculosis* (Mtb) detection and drug susceptibility testing (DST) (1). Culture’s sensitivity and usefulness in diagnostics and treatment response monitoring depend on sample quality (2, 3). Culture contamination may lead to false negative or indeterminate results, increasing the burden on healthcare systems when repeated sample collection and testing are required and potentially delaying treatment initiation (4–7). Colony-forming units (CFUs) and time to positivity (TTP), measures associated with TB disease burden and level of infectiousness, and early bactericidal activity (EBA) for anti-TB medication assessments may be inaccurate and uninterpretable in contaminated samples (8–10). Additionally, contaminant DNA affects culture results and introduces false genetic variability in sequencing, including emerging methods for detecting drug resistance and strain diversity without the biohazard risks of traditional culture methods (11).

Despite this, there is limited consensus on optimal collection, storage, transport, and handling practices to minimize contamination, and current protocols rarely assess the sputum quality for TB diagnostics (12, 13). The Stop TB Partnership and World Health Organization (WHO) recommend at least one specimen be collected in the early morning, with strict adherence to standardized laboratory protocols (14), including refrigeration at 2–8°C within 1 h of collection and processing within 48 h (15). In practice, procedures are site- and/or laboratory-specific (16, 17). As smoking cigarettes and eating can alter the lung and oral microbiome, an oral wash before collection is suggested to improve sample quality (5, 18–24), as well as pooling specimens, various storage conditions, shortening transport intervals, and providing patient instruction and supervising collection, of which the latter has shown the most promise (13, 24–26).

The United States Center for Disease Control (CDC) proposes a 3–5% contamination target as an acceptable benchmark for a well-functioning biosafety level 3 (BSL-3) diagnostic facility (27). Achieving these standards in low- to middle-income country (LMIC) settings is often challenging due to structural and financial barriers. In LMICs, BSL-3 facilities are typically centralized in national reference laboratories or tertiary care hospitals (28). Sputum specimens often require long-distance transport to reach these centralized locations, and maintaining the recommended cold chain is not always feasible. Information on how these delays impact contamination risk could identify regions where stricter protocols could improve diagnostic accuracy.

We aimed to evaluate rates and predictors of contamination in serially collected sputum specimens from individuals undergoing treatment for drug-susceptible pulmonary TB. Specifically, we examined the relationships between contamination, supervised specimen collection, and the time elapsed from sputum collection to MGIT inoculation during the first 12 weeks of treatment. Additionally, we assessed associations between sociodemographic, substance use, sputum quality, and clinical variables.

## MATERIALS AND METHODS

### Study setting and participants

The Tuberculosis Treatment and Alcohol Use Study (TRUST) was a prospective longitudinal cohort that enrolled individuals initiating TB treatment to assess the impact of alcohol consumption on treatment response (29). Inclusion criteria were age ≥ 15 years, Mtb bacterial confirmation (positive GeneXpert MTB/RIF or Ultra and/or mycobacterial culture) without evidence of rifampicin resistance, not pregnant at enrollment, and living in Worcester, South Africa (29). Participants consented to an enrollment visit on the day of treatment initiation, daily directly observed therapy (DOT) during the 6-month treatment period, and post-treatment visits up to 1 year after treatment completion. Participants were assigned a study DOT worker and followed during treatment, with sputum specimens collected weekly for 12 weeks post-treatment initiation and at the 5-month follow-up visit (30). For this analysis, participants were excluded if no treatment initiation (week 1) sputum specimen was collected. Ethical approval was obtained from the Boston Medical Center Institutional Review Board (H-34970), the Health Research Ethics Committees from the South African Medical Research Council (EC011-5/2016), and Stellenbosch University (N17/04/039 RECIP_MRC_EC011-5/2016).

### Sputum collection procedures

Sputum specimens were considered collected under supervision if produced in the presence of a field team member (research nurse, trained staff, or DOT worker). Supervised collection involved the field team member guiding the collection, including mouth rinse, keeping the specimen jar closed until expectoration, and ensuring a good-quality sputum (color, viscosity, and volume). Specimens were classified as unsupervised if participants produced them at home, and then brought the sample to the research site (week 1 sample) or handed it to the DOT worker (daily visits at weeks 2–12). Participants were instructed to follow procedures used during supervised collections and produce sputum specimens first in the morning. However, we found that they often delayed collection until the DOT worker arrived (therefore resulting in supervised collection).

**Fig 1:**
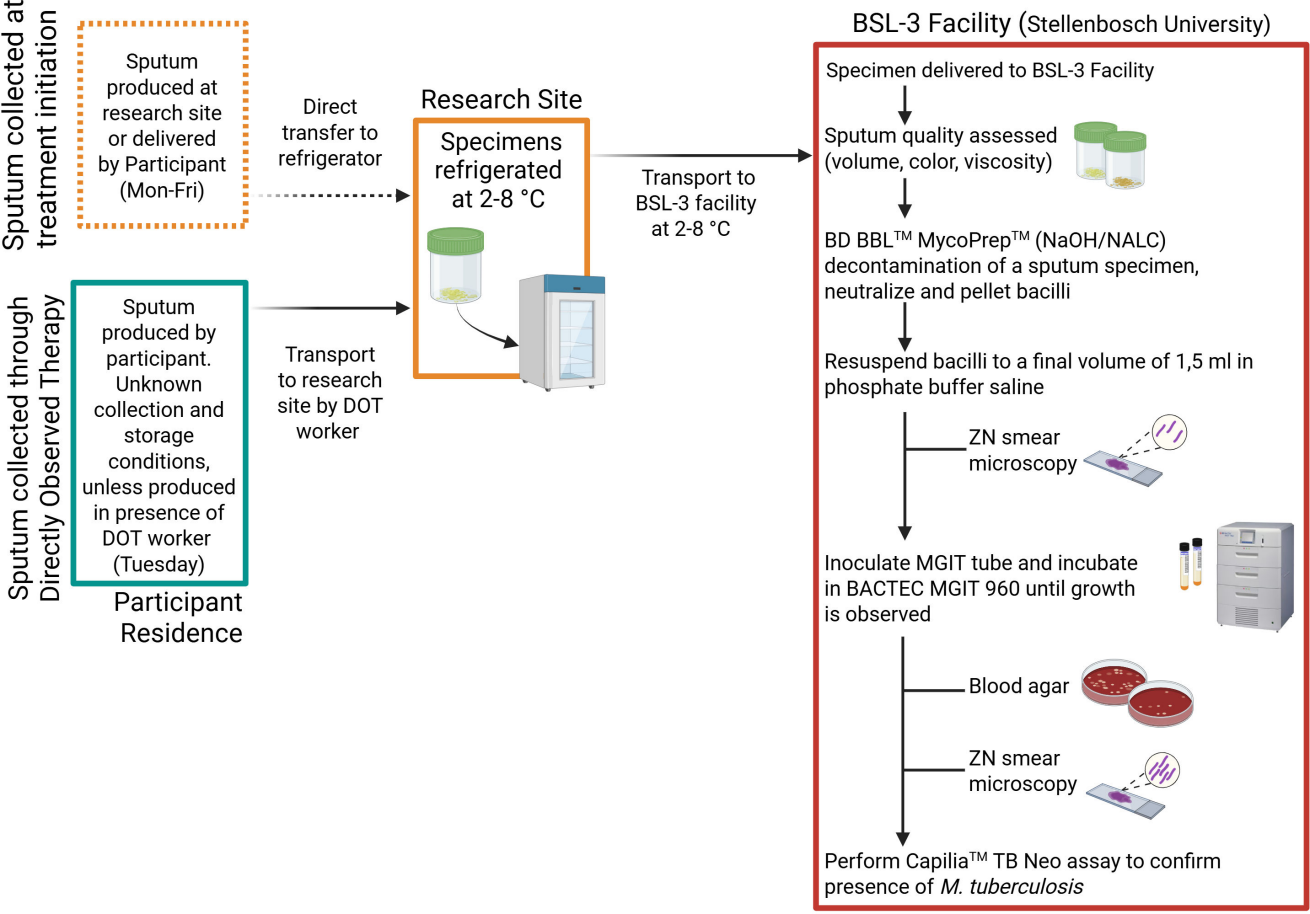
Sputum specimen collection and analysis strategy. Flow diagram illustrating the protocol for sputum specimen collection over the first 12 weeks of treatment initiation, interim storage conditions, and transport. The protocol followed within the BSL-3 facility for *M. tuberculosis* culture in mycobacterial growth indicator tubes for this study is outlined within the red rectangle. Created in BioRender. Warren, R. (2025) https://BioRender.com/7417p11

For the treatment initiation visit (referred to as week 1), specimens collected at the research site or delivered by the participant were immediately refrigerated at 2–8°C (Fig. 1). Throughout weeks 2–12, DOT workers collected all sputum specimens on Tuesdays. Participants were asked to keep specimens cool until DOT workers collected them. Specimens were stored in cooler boxes with ice packs by the DOT workers until delivered to the research site for refrigeration. Sputum specimens were refrigerated at 2–8°C until twice-weekly transport (Wednesday and Friday) in a cooler box with ice packs to the BSL-3 facility at Stellenbosch University, Cape Town (approximately 1-h drive), maintaining the cold chain and in compliance with WHO guidelines (Fig. 1) (14). No on-site decontamination was performed before transportation to the BSL-3 facility.

### Specimen processing

Sputum quality (volume, color, viscosity) was recorded before decontamination with the BBL MycoPrep Specimen Digestion/Decontamination Kit as per the manufacturer’s instructions (Fig. 1). Briefly, sputum was processed by adding 2% sodium hydroxide (NaOH) with 0.5% N-acetyl-L-cysteine (NALC) with a final concentration of 1% NaOH. The specimen/NALC-NaOH mixtures were vortexed until a homogeneous sample was obtained, whereafter it was allowed to stand at ambient temperature for 15 min with occasional gentle inversion. Processing was completed by adding a phosphate buffer saline (PBS; pH 6.8) and centrifugation (15 min at 3,000 × *g*). Concentrated pellets were resuspended to a volume of 1,5 mL in PBS. Ziehl-Neelsen (ZN) smear microscopic analysis was performed on all decontaminated crude sediments to evaluate and grade the presence of acid-fast bacilli (AFB). In addition, all sediments (0.5 mL) were inoculated into Mycobacteria Growth Indicator Tube 960 (MGIT, Becton Dickinson Microbiology Systems, USA) containing polymyxin B, amphotericin B, nalidixic acid, trimethoprim, and azlocillin (PANTA, BD, USA) and incubated in the BACTEC MGIT 960. Once a growth index of 70 growth units was observed, TTP was automatically recorded on the MGIT, and contamination was assessed by ZN smear microscopy and blood agar culture. All MGIT samples with a delayed TTP (longer than 20 days) with an AFB-positive ZN smear were subjected to immune-chromatographic analysis, namely, Capilia TB assay (TAUNS Laboratories, Inc.), to confirm the presence of Mtb versus non-tuberculous mycobacteria (NTM).

In this study, specimens were considered contaminated if a positive MGIT culture was observed to contain contaminant growth on either the culture ZN smear and/or blood agar (regardless of the presence of Mtb). This differs from the definition used within diagnostic laboratories, including the South African National Health Laboratory Services (NHLS), which considers specimens contaminated only if a positive MGIT culture was observed to contain growth, but no Mtb on the ZN smear (referred to as diagnostic definition) (31, 32).

### Measures

At the treatment initiation visit, sociodemographic (age, race, biological sex, etc.) and substance use information were collected. Harmful alcohol use was defined as a phosphatidylethanol (PEth) blood test > 49 ug/L and/or self-report with an Alcohol Use Disorders Identification Test (AUDIT) score > 7 (33). The Fagerström Test for Nicotine Dependence (FTND) was administered to screen for current tobacco use, while smoked drug use was defined by a positive urine drug test or self-reported use of methaqualone, methamphetamine, and/or cannabis (34). ZN smear is graded no AFB, scanty, 1+, 2+, and 3+ (15).

Chest radiographs captured lung cavitation, while clinical data on HIV status, previous TB disease, and other comorbidities were extracted from medical charts. The treatment week variable was defined as the week since treatment initiation (week 1). Time to culture was defined as the interval in days between specimen collection and MGIT inoculation.

### Statistical analysis

Contamination rates were calculated weekly as the percentage of contaminated specimens and stratified by AFB presence. The percentage of AFB-positive samples without contamination and that of samples with no growth (Mtb nor contaminants) were calculated after 42 days.

Associations between contamination at treatment initiation and collection type (supervised versus unsupervised), time to culture (2 days or <2 days), sociodemographic factors, substance use, sputum quality, and clinical variables were examined using simple logistic regression models. Any variables associated with contamination (*P*-value ≤ 0.1) were included in the multivariate analysis. Missing observations for each variable are noted within Table 1. One participant’s HIV status is missing due to inability to collect blood. Sputum volume, culture, and time to culture variables were not captured for one specimen. Two radiographs were missing at treatment initiation due to a participant being hospitalized and another having a poor-quality film, while five readings were not able to confirm the presence of cavitary TB disease. The supervised expectoration variable was missing for 25 samples at treatment initiation (week 1) due to lack of recording.

**TABLE 1:** Bivariate associations^*a*^

	Median (IQR) or frequency (%)			Odds ratio (95% CI)	*P*-value^*c*^
	Total *n* = 301	Contaminated^*b*^ *n* = 37	Non-contaminated *n* = 264		
Male (sex)	180 (59.8)	18 (48.6)	162 (61.4)	0.60 (0.30, 1.19)	0.143
Age (years, ref. <30)					0.990
<30	87 (28.9)	10 (27.0)	77 (29.2)		
30–39	77 (25.6)	10 (27.0)	67 (25.4)	1.15 (0.45, 2.96)	
40–49	73 (24.3)	9 (24.3)	64 (24.2)	1.08 (0.41, 2.85)	
>50	64 (21.3)	8 (21.6)	56 (21.2)	1.10 (0.40, 2.96)	
Living with HIV (*n* = 300)	85 (28.3)	11 (29.7)	74 (28.1)	1.08 (0.49, 2.25)	0.840
Tobacco use	205 (68.1)	21 (56.8)	184 (69.7)	0.57 (0.28, 1.17)	0.117
Smoked drug use^*d*^	165 (54.8)	15 (40.5)	150 (56.8)	0.52 (0.25, 1.04)	0.066
Harmful alcohol use^*e*^	186 (61.8)	23 (62.2)	163 (61.7)	1.02 (0.51, 2.11)	0.961
Sputum volume (mL) (*n* = 300)	2 (1,4)	3 (2,5)	2 (1,4)	1.13 (0.97, 1.29)	0.093
Sputum color (yes) (*n* = 300)	244 (81.3)	28 (75.7)	216 (82.1)	0.68 (0.31, 1.61)	0.348
Supervised expectoration (*n* = 276)	263 (95.3)	25 (86.2)	238 (96.4)	0.24 (0.07, 0.92)	0.024
Time to culturing ≥ 2 days^*f*^ (*n* = 300)	142 (47.3)	21 (56.8)	121 (46.0)	1.54 (0.77, 3.13)	0.223
Smear grade (ref. +++)^*g*^					0.290
+++	85 (28.2)	10 (27.0)	75 (28.4)		
++	48 (15.9)	3 (8.1)	45 (17.0)	0.50 (0.06, 0.25)	
+	48 (15.9)	10 (27.0)	38 (14.4)	1.97 (0.11, 1.74)	
Scanty	32 (10.6)	3 (8.1)	29 (11.0)	0.76 (0.17, 2.75)	
No AFB^*h*^	88 (29.2)	11 (29.7)	77 (29.2)	1.07 (0.43, 2.71)	
Culture-positive^*i*^	274 (91.0)	33 (89.2)	241 (91.3)	0.79 (0.28, 2.81)	0.676
Cavitary TB disease^*j*^ (*n* = 294)	191 (63.7)	22 (59.5)	169 (64.3)	0.83 (0.41, 1.73)	0.605

^*a*^ Associations between sputum contamination in samples collected during treatment initiation visit (week 1 sputum specimen) and demographic, clinical, and collection condition variables. *n* = 301.

^*b*^ Presence of contaminants on a blood agar plate or smear microscopy after detection of a positive MGIT culture.

^*c*^ All *P*-values represented were calculated using simple logistic regression models.

^*d*^ Defined as a positive urine drug test or self-reported use of methaqualone, methamphetamine, and/or cannabis.

^*e*^ Defined as a phosphatidylethanol (PEth) blood test > 49 ug/L and/or an AUDIT score > 7.

^*f*^ Time from collection to processing and MGIT inoculation.

^*g*^ Observed with concentrated sputum smear microscopy.

^*h*^ Acid fast bacilli.

^*i*^ Presence of confirmed *M. tuberculosis*.

^*j*^ Lung cavitation identified using chest X-ray.

We fitted a multivariate logistic model with contamination as the outcome adjusting for age, sex, HIV status, smoked drug use, collection type (supervised vs. unsupervised), and time to culture. Although age, sex, and HIV status were not associated with contamination (*P*-value > 0.1), they were included in the model due to their potential impact on the specimen quality (35–40).

To examine longitudinal associations, we fitted a logistic regression model using generalized estimating equations (GEE) to account for within-participant correlation of samples collected over time. We adjusted for age, sex, HIV status, and smoked drug use. Smear grade and collection type were included as time-varying covariates, with smear grade included to account for declining bacillary burden during treatment. We also adjusted for treatment week of the sputum collection. Accordingly, 0.6% of samples (*N* = 15) were stored for ≥2 days before being processed. Due to the low variability in this measure, it was not included in the longitudinal model. A total of 214 observations (6.8%) were excluded from the longitudinal model due to lack of collection of the supervision variable in the first 6 months of the study.

All statistical tests were assessed using a 0.05 significance level. Analyses were conducted using R version 3.6.2.

## RESULTS

Between May 2017 and May 2022, 3,155 sputum specimens were collected from 301 participants who were diagnosed with pulmonary Mtb (positive GeneXpert MTB/RIF or Ultra and/or mycobacterial culture). A total of 301 samples were collected at treatment initiation visits and 2,854 specimens across the remaining 11-week sampling period. At treatment initiation (week 1), 213 (70.8%) sputum specimens were smear-positive (grade scanty or above), and 274 (91.0%) were Mtb culture-positive. The median participant age was 38 years (IQR 27,48); 180 participants (59.8%) were male, and 85 (28.3%) were living with HIV (PLWH) (Table 1).

### Rates of contamination over time

**Fig 2:**
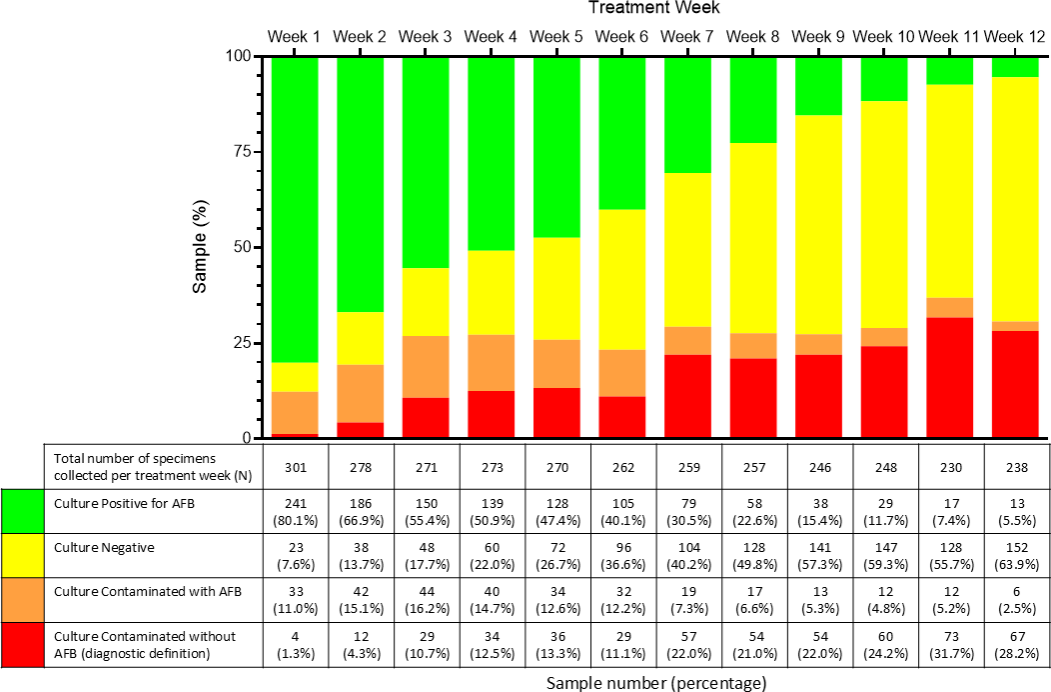
Contamination rates for 12 weeks following TB treatment initiation. *N* = 3155. Green bars represent the percentage of AFB-positive samples with no contamination, and yellow bars indicate samples with no growth after 42 days. Orange bars indicate the percentage of samples contaminated in the presence of AFB. Red bars indicate the percentage of samples contaminated in the absence of AFB (diagnostic definition). The table indicates the total number of samples collected per treatment week, as well as the number and percentage of culture-positive or negative samples for the respective week following treatment initiation with and without contamination.

**Fig 3:**
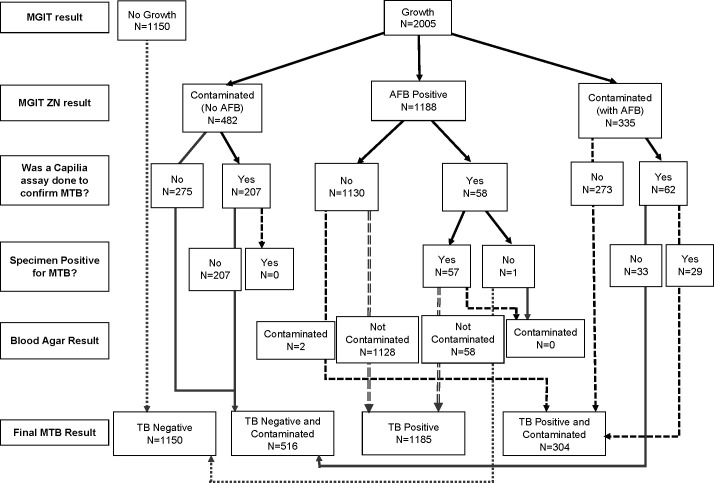
Classification of sample contamination. The flow diagram illustrating the classification of sample contamination after the BACTEC MGIT 960 system flags a culture positive for growth. If the MGIT culture is not flagged as positive after 42 days of incubation in the BACTEC MGIT 960 system, it is regarded as culture-negative (no growth). MGIT = mycobacterial growth indicator tubes; ZN = Zeihl neelsen; AFB = acid fast bacilli; Mtb = *M. tuberculosis*.

Contamination rates varied over the first 12 weeks of treatment, ranging from 12.3% (37/301) of week 1 (treatment initiation) samples to a maximum of 36.9% (85/232) of week 11 samples (Fig. 2). Of the 355 MGIT cultures with AFB and contamination according to ZN smear, 62 (17.5%) underwent confirmatory Capilia testing, resulting in 29 (46.8%) samples detecting Mtb antigen (Fig. 3). When applying the diagnostic definition for contamination used within laboratories, such as NHLS, contamination rates ranged from 1.3% (4/301) for week 1 samples to 31.7% (73/232) for week 11 samples (Fig. 2) (32). All contaminated samples per the diagnostic definition were culture-negative for Mtb and no AFB were observed on smear microscopy.

### Predictors of contamination at treatment initiation

At treatment initiation, supervised collection was associated with significantly less contamination (unadjusted odds ratio [OR] = 0.24, 95% confidence interval [CI] 0.07–0.92, *P* = 0.024) (Table 1). Although not statistically significant, samples cultured 2 days or longer after collection (142/300) were more frequently contaminated (56.8% vs 46.0%, *P* = 0.223).

The specimen color, cavitary TB disease, and culture positivity were not associated with contamination (Table 1). Specimen volume, although marginally associated with contamination (*P* = 0.093), was not included in the adjusted model due to concerns about its reliability. Harmful alcohol use and tobacco use were not associated with contamination rates, while the association between smoked drug use and contamination approached significance (OR 0.52, 95% CI 0.25–1.04, *P* = 0.066) (Table 1). When applying the diagnostic definition, only four samples were considered contaminated (Table S1). All contaminated samples were culture-negative for Mtb, and no AFB were observed on smear microscopy.

A total of 274 sputum samples (27 excluded due to missing covariate data) were included in the adjusted analyses of contamination at treatment initiation. Age, sex, HIV status, smoked drug use, and time to culture were not associated with contamination rates. There was a significant association of supervised collection with decreased contamination, with the odds of contamination consistent with unadjusted analysis (OR = 0.26, 95% CI 0.07–1.10, *P* = 0.048) (Table 2).

**TABLE 2:** Multivariate associations at treatment initiation^*a*^

	OR^*b*^	95% CI^*c*^	*P*-value
Supervised expectoration	0.26	0.07, 1.10	0.048
Time to culture, ≥2 days^*d*^	1.58	0.71, 3.59	0.267
Age (years, ref. <30)			
30–39	1.14	0.38, 3.55	0.813
40–49	0.84	0.25, 2.75	0.770
>50	1.06	0.32, 3.50	0.923
Smoked drug use	0.48	0.20, 1.13	0.098
Male (sex)	1.07	0.46, 2.54	0.882
HIV positive	1.28	0.51, 3.00	0.587

^*a*^ A multivariate logistic model fit with contamination as the outcome and adjusting for time between sputum collection and processing, sex, age, smoked drug use, HIV status, and supervision of specimen collection. *N* = 274.

^*b*^ Odds ratio.

^*c*^ Confidence interval.

^*d*^ Time from collection to processing and MGIT inoculation.

### Predictors of contamination across the 12-week sampling period

The model evaluating predictors of contamination over time included 2,915 sputum samples (Table 3); 218 samples were excluded due to missing covariate data. Over time, the supervised collection remained a significant predictor of reduced contamination (OR = 0.79, 95% CI 0.64–0.98, *P* = 0.031). A negative or scanty sputum smear grade was associated with a higher risk of contamination (OR = 1.55, 95% CI 1.05–2.29, *P* = 0.028) compared to a smear grade of 3+ (OR = 1.99, 95% CI 1.31–3.03, *P* = 0.001). Age, sex, HIV status, time to culture, and smoked drug use were not associated with contamination. A significant association was found between contamination and the treatment week of sputum collection, with each additional week increasing the odds of contamination (OR = 1.07, 95% CI 1.03–1.10, *P* < 0.001). In contrast, when using the diagnostic definition (Table S2), supervised expectoration was not significantly associated with contamination (*P* = 0.214). Treatment week (OR: 1.21; CI 1.18–1.25, *P* < 0.001) continued to be significantly associated with contamination, with the addition of HIV positivity emerging as an additional significant predictor (OR: 1.49; CI 1.13, 1.96, *P* = 0.005).

**TABLE 3:** Longitudinal associations across the 12 treatment weeks^*a*^

	OR^*b*^	95% CI^*c*^	*P*-value
Supervised expectoration	0.79	0.64, 0.98	0.031
Smear grade (ref. +++)^*d*^			
No AFB^*e*^	1.55	1.05, 2.29	0.028
Scanty	1.99	1.31, 3.03	0.001
+	1.39	0.93, 2.09	0.107
++	1.35	0.83, 2.20	0.223
Age (years, ref. <30)			
30–39	0.97	0.71, 1.32	0.835
40–49	0.91	0.66, 1.25	0.553
>50	0.96	0.69, 1.34	0.804
Smoked drug use	1.06	0.83, 1.36	0.642
Male (sex)	1.02	0.80, 1.31	0.876
Living with HIV	1.14	0.90, 1.44	0.278
Treatment week^*f*^	1.07	1.03, 1.10	<0.001

^*a*^ A logistic regression model was fitted to account for the within-participant correlation of contamination status from treatment initiation up to treatment week 12. *N* = 2915.

^*b*^ Odds ratio.

^*c*^ Confidence interval.

^*d*^ Observed with concentrated sputum smear microscopy.

^*e*^ Acid fast bacilli.

^*f*^ Timepoint at which sample was collected by field or DOTS workers across a 12-week sampling period following treatment initiation.

## DISCUSSION

Despite advances in sputum-based TB diagnostics, culture contamination remains a significant issue leading to false negatives, indeterminate results, and unreliable analytic outcomes. Therefore, quality specimens are essential, and rigorous collection, storage, and transport protocols are required to ensure the optimal use of resources.

Our results indicate a large increase in culture contamination in our study from week 1 (12.3%) to week 2 (19.3%) likely due to the change from clinic to field-based sampling with less supervision. Although specimen collection, transport, and processing procedures remained constant from week 2 onwards, contamination rates further increased in week 3 and remained relatively steady for the rest of the 12 week sampling period (23.3–36.9%). This aligns with previous research reporting contamination rates in TB diagnostic samples ranging from 0% at treatment initiation to 37% in follow-up samples (25, 26, 41). This is likely due to the reduced Mtb bacilli burden, allowing other microbes to grow while the sample processes for longer and introduced oral and esophageal flora as sputum specimens become more difficult to produce in later treatment weeks. Our findings support this explanation, with increased odds of contamination among samples with a negative or scanty smear result compared to 3+ sputum smear microscopy grade and a significant association between the treatment week of specimen collection and contamination.

It is important to note that in the South African NHLS, contamination is defined as growth-positive MGIT culture with no AFB by ZN microscopy (31, 32). Samples are only used for diagnostic purposes, as opposed to research laboratories, where downstream use of the Mtb isolates is essential, and data are only interpretable if not contaminated. Our definition, therefore, considers a sample contaminated if other microbes are present on either ZN stain or blood agar, regardless of Mtb growth. Using the diagnostic laboratory definition, the contamination rate at treatment initiation in this study would be 1.3%, well below the proposed 3–5% (27).

In 62 MGIT cultures with AFB and contamination that underwent confirmatory Capilia testing, only 29 cases contained Mtb antigen, demonstrating the presence of non-tuberculous mycobacteria (NTM) frequently seen in patients receiving anti-TB treatment (42). Since NTM are ubiquitous environmental organisms, these NTM-positive cultures may indicate colonization or infection (43).

Supervised sputum collection reduced contamination rates both at treatment initiation and in serial samples over the first 12 weeks of TB treatment. Previous reports have suggested contamination rates of 37% observed among samples collected at home without supervision (26). Supervised collection was associated with a 74% reduction in the odds of contamination for treatment initiation samples. In the longitudinal analysis, it was also linked to a 21% reduction in the odds of contamination, demonstrating its effectiveness in reducing contamination, even in later-in-treatment samples. Contamination rates in weeks 8–12 ranged from 27.6 to 36.9%, but supervised collection and proper instruction likely helped mitigate contamination, particularly as the bacillary burden decreased over time.

At treatment initiation, samples inoculated for MGIT culture 2 days or longer after collection were observed to be slightly more frequently contaminated (56.8%). The lack of significance may reflect the small number of samples with delayed inoculation (47.3%). Our findings support that aiming for inoculation within 2 days of sample collection should yield reliable, low contamination rates and are consistent with a South African study that found no significant association between contamination and time from collection to processing (0–4 days) (44, 45). However, previous studies have reported increased contamination with longer storage times (46, 47). We were unable to assess the impact of delayed cold chain on contamination for samples collected during weeks 2–12, as there was little variability in the time from collection to culture. Maintaining a strict cold chain, therefore, seems crucial for samples collected later in treatment, when bacillary burden and sputum quality may decline.

No significant association was found with any substance use. In unadjusted analyses, smoked drug use appeared to have a protective effect; however, these results may be confounded by bacterial load, as higher bacterial load, more common among people who use drugs, may reduce the likelihood of contamination. This is supported by longitudinal models adjusted for smear grade, where the association was in the expected direction, although not significant.

Finally, the Stop TB Partnership and WHO guidelines specify that at least one specimen for Mtb testing be collected early morning to reduce contamination; however, in practice, this may be challenging (1). One limitation is that DOT workers did not consistently capture when and under what conditions unsupervised specimen collections occurred. In addition, participants were asked to store specimens in a refrigerator after the morning expectoration, but since many participants did not have access to refrigerators, this level of detail was not captured.

### Conclusion

Measures like Mtb culture positivity and analytic measures (e.g., TTP, DNA sequencing) can be inaccurate and uninterpretable when sputum cultures are contaminated. Our study benefits from serial sputum sampling over the first 12 weeks of TB treatment, allowing us to assess contamination rates while accounting for the decline in bacillary burden throughout treatment. This approach is novel, as other studies typically only report contamination data at baseline and treatment completion. Our findings emphasize the benefits of supervised sputum collection for TB diagnosis and monitoring treatment outcomes and show that while contamination rates generally increase in later-treatment samples, supervised collection can help mitigate this issue. These results can inform decentralized TB monitoring programs, emphasizing the importance of participant education and supervision during sample collection to enhance diagnostic accuracy and treatment effectiveness in a high-burden, LMIC setting.

## SUPPLEMENTAL MATERIAL

Tables S1 and S2 spectrum.00969-25-s0001.pdf Stratified descriptive table and longitudinal model using the diagnostic laboratory definition.

ASM does not own the copyrights to Supplemental Material that may be linked to, or accessed through, an article. The authors have granted ASM a non-exclusive, world-wide license to publish the Supplemental Material files. Please contact the corresponding author directly for reuse.

## References

[1] World Health Organization. 2020. Global tuberculosis report 2020. Geneva.

[2] Banda HT, Harries AD, Boeree MJ, Nyirenda TE, Banerjee A, Salaniponi FM. 2000. Viability of stored sputum specimens for smear microscopy and culture. Int J Tuberc Lung Dis 4:272–274. https://europepmc.org/article/MED/10751076. 10751076

[3] Zerbini MS, Singh S, Botha M, Ghebrekristos Y, Opperman CJ. 2023. Specimen rejection in a high-throughput TB laboratory: a descriptive study. S Afr Med J 113:6–7. doi:10.7196/SAMJ.2023.v113i10.1364 37881905

[4] Muzanye G, Morgan K, Johnson J, Mayanja-Kizza H. 2009. Impact of mouth rinsing before sputum collection on culture contamination. Afr Health Sci 9:200. 20589151 PMC2887023

[5] Kalema N, Boon SD, Cattamanchi A, Davis JL, Andama A, Katagira W, Everett C, Walter N, Byanyima P, Kaswabuli S, Worodria W, Huang L. 2012. Oral antimicrobial rinse to reduce mycobacterial culture contamination among tuberculosis suspects in Uganda: a prospective study. PLoS One 7:e38888. doi:10.1371/journal.pone.0038888 22808020 PMC3395623

[6] de Boer AS, Blommerde B, de Haas PEW, Sebek MMGG, Lambregts-van Weezenbeek KSB, Dessens M, van Soolingen D. 2002. False-positive Mycobacterium tuberculosis cultures in 44 laboratories in The Netherlands (1993 to 2000): incidence, risk factors, and consequences. J Clin Microbiol 40:4004–4009. doi:10.1128/JCM.40.11.4004-4009.2002 12409366 PMC139647

[7] Asgharzadeh M, Ozma MA, Rashedi J, Poor BM, Agharzadeh V, Vegari A, Shokouhi B, Ganbarov K, Ghalehlou NN, Leylabadlo HE, Kafil HS. 2020. False-positive Mycobacterium tuberculosis detection: ways to prevent cross-contamination. Tuberc Respir Dis (Seoul) 83:211–217. doi:10.4046/trd.2019.0087 32578410 PMC7362751

[8] Olaru ID, Heyckendorf J, Grossmann S, Lange C. 2014. Time to culture positivity and sputum smear microscopy during tuberculosis therapy. PLoS One 9:e106075. doi:10.1371/journal.pone.0106075 25171337 PMC4149502

[9] Sloan DJ, Corbett EL, Butterworth AE, Mwandumba HC, Khoo SH, Mdolo A, Shani D, Kamdolozi M, Allen J, Mitchison DA, Coleman DJ, Davies GR. 2012. Optimizing outpatient serial sputum colony counting for studies of tuberculosis treatment in resource-poor settings. J Clin Microbiol 50:2315–2320. doi:10.1128/JCM.00043-12 22573593 PMC3405637

[10] Ellappan K, Datta S, Muthuraj M, Lakshminarayanan S, Pleskunas JA, Horsburgh CR Jr, Salgame P, Hochberg N, Sarkar S, Ellner JJ, Roy G, Jose M, Vinod Kumar S, Joseph NM. 2020. Evaluation of factors influencing Mycobacterium tuberculosis complex recovery and contamination rates in MGIT960. Indian J Tuberc 67:466–471. doi:10.1016/j.ijtb.2020.07.016 33077045

[11] Mann BC, Loubser J, Omar S, Glanz C, Ektefaie Y, Jacobson KR, Warren RM, Farhat MR. 2024. Systematic review and meta-analysis of protocols and yield of direct from sputum sequencing of Mycobacterium tuberculosis. bioRxiv:2024.12.04.625621. doi:10.1101/2024.12.04.625621

[12] Ho J, Marks GB, Fox GJ. 2015. The impact of sputum quality on tuberculosis diagnosis: a systematic review. Int J Tuberc Lung Dis 19:537–544. doi:10.5588/ijtld.14.0798 25868021

[13] Datta S, Shah L, Gilman RH, Evans CA. 2017. Comparison of sputum collection methods for tuberculosis diagnosis: a systematic review and pairwise and network meta-analysis. Lancet Glob Health 5:e760–e771. doi:10.1016/S2214-109X(17)30201-2 28625793 PMC5567202

[14] World Health Organization. 2000. Regional office for south-east Asia. Guidelines on standard operating procedures for microbiology. WHO Regional Office for South-East Asia

[15] Stop TB Partnership. 2013. Laboratory diagnosis of tuberculosis by sputum microscopy – The GLI handbook. Global Laboratory Initiative. https://www.stoptb.org/laboratory-diagnosis-tuberculosis-sputum-microscopy-gli-handbook.

[16] Tuberculosis Coalition for Technical Assistance. 2006. International Standards for Tuberculosis Care (ISTC. Tuberculosis Coalition for Technical Assistance, The Hague. https://www.who.int/publications/m/item/international-standards-for-tuberculosis-care-%28istc%29.

[17] Lumb R, Deun A, Bastian I, Fitz-gerald M. 2013. Laboratory diagnosis of tuberculosis by sputum microscopy - the handbook global edition

[18] Huang C, Shi G. 2019. Smoking and microbiome in oral, airway, gut and some systemic diseases. J Transl Med 17:225. doi:10.1186/s12967-019-1971-7 31307469 PMC6632217

[19] Yang Y, Yu X, Yang X, Zeng K, Liu G, Hao W, Zhang S, Wang G. 2021. Oral microbiota profile of individuals who abuse methamphetamine. Front Cell Infect Microbiol 11. doi:10.3389/fcimb.2021.706961 PMC8461105 34568092

[20] Lee HH, Sudhakara P, Desai S, Miranda K, Martinez LR. 2021. Understanding the basis of METH mouth using a rodent model of methamphetamine injection, sugar consumption, and Streptococcus mutans Infection. mBio 12:e03534-20. doi:10.1128/mBio.03534-20 33688011 PMC8092307

[21] Wu J, Peters BA, Dominianni C, Zhang Y, Pei Z, Yang L, Ma Y, Purdue MP, Jacobs EJ, Gapstur SM, Li H, Alekseyenko AV, Hayes RB, Ahn J. 2016. Cigarette smoking and the oral microbiome in a large study of American adults. ISME J 10:2435–2446. doi:10.1038/ismej.2016.37 27015003 PMC5030690

[22] Jia YJ, Liao Y, He YQ, Zheng MQ, Tong XT, Xue WQ, Zhang JB, Yuan LL, Zhang WL, Jia WH. 2021. Association between oral microbiota and cigarette smoking in the Chinese population. Front Cell Infect Microbiol 11:658203. doi:10.3389/fcimb.2021.658203 34123872 PMC8195269

[23] Peres RL, Palaci M, Loureiro RB, Dietze R, Johnson JL, Golub JE, Ruffino-Netto A, Maciel EL. 2011. Evaluation of oral antiseptic rinsing before sputum collection to reduce contamination of mycobacterial cultures. J Clin Microbiol 49:3058–3060. doi:10.1128/JCM.00541-11 21677070 PMC3147721

[24] Kabore A, Tranchot-Diallo J, Sanou A, Hien H, Daneau G, Gomgnimbou MK, Meda N, Sangaré L. 2019. Why oral antiseptic mouth rinsing before sputum collection cannot reduce contamination rate of mycobacterial culture in Burkina-Faso. Afr Health Sci 19:1321–1328. doi:10.4314/ahs.v19i1.3 31148957 PMC6531976

[25] Muzanyi G, Angel M, Nakamate T, Ogwang S, Nyole S. 2011. Impact of directly observed sputum collection on sputum culture contamination rates. Afr Health Sci 11:605–606. 22649442 PMC3362976

[26] Maciel ELN, Prado TN do, Peres RL, Palaci M, Johnson JL, Dietze R. 2009. Guided sputum sample collection and culture contamination rates in the diagnosis of pulmonary TB. J Bras Pneumol 35:460–463. doi:10.1590/s1806-37132009000500012 19547856

[27] Kent PT, Kubica GP. 1985. Public health mycobacteriology: a guide for the level II laboratory. Available from: https://ntrl.ntis.gov/NTRL/dashboard/searchResults/titleDetail/PB86216546.xhtml

[28] Harries AD, Kumar AMV. 2018. Challenges and progress with diagnosing pulmonary tuberculosis in low- and middle-income countries. Diagnostics (Basel) 8:78. doi:10.3390/diagnostics8040078 30477096 PMC6315832

[29] Myers B, Bouton TC, Ragan EJ, White LF, McIlleron H, Theron D, Parry CDH, Horsburgh CR, Warren RM, Jacobson KR. 2018. Impact of alcohol consumption on tuberculosis treatment outcomes: a prospective longitudinal cohort study protocol. BMC Infect Dis 18:488. doi:10.1186/s12879-018-3396-y 30268101 PMC6162918

[30] Ragan EJ, Gill CJ, Banos M, Bouton TC, Rooney J, Horsburgh CR, Warren RM, Myers B, Jacobson KR. 2021. Directly observed therapy to measure adherence to tuberculosis medication in observational research: protocol for a prospective cohort study. JMIR Res Protoc 10:e24510. doi:10.2196/24510 34132642 PMC8277341

[31] Ghebrekristos Y, Beylis N, Centner C, Venter R, Derendinger B, Tshivhula H, Naidoo S, Alberts R, Prins B, Tokota A, Dolby T, Marx F, Omar S, Warren R, Theron G. 2022. Something from nothing: sensitivity and specificity of Xpert MTB/RIF ultra on contaminated liquid cultures for tuberculosis and rifampicin-resistance detection. medRxiv. doi:10.1101/2022.12.07.22283223 PMC10600950 37739001

[32] Ghebrekristos YT, Beylis N, Centner CM, Venter R, Derendinger B, Tshivhula H, Naidoo S, Alberts R, Prins B, Tokota A, Dolby T, Marx F, Omar SV, Warren R, Theron G. 2023. Xpert MTB/RIF ultra on contaminated liquid cultures for tuberculosis and rifampicin-resistance detection: a diagnostic accuracy evaluation. Lancet Microbe 4:e822–e829. doi:10.1016/S2666-5247(23)00169-6 37739001 PMC10600950

[33] Bohn MJ, Babor TF, Kranzler HR. 1995. The alcohol use disorders identification test (AUDIT): validation of a screening instrument for use in medical settings. J Stud Alcohol 56:423–432. doi:10.15288/jsa.1995.56.423 7674678

[34] Heatherton TF, Kozlowski LT, Frecker RC, Fagerström KO. 1991. The fagerström test for nicotine dependence: a revision of the fagerström tolerance questionnaire. Br J Addict 86:1119–1127. doi:10.1111/j.1360-0443.1991.tb01879.x 1932883

[35] Elliott AM, Namaambo K, Allen BW, Luo N, Hayes RJ, Pobee JO, McAdam KP. 1993. Negative sputum smear results in HIV-positive patients with pulmonary tuberculosis in Lusaka, Zambia. Tuber Lung Dis 74:191–194. doi:10.1016/0962-8479(93)90010-U 8369514

[36] Joloba ML, Johnson JL, Namale A, Morrissey A, Assegghai AE, Mugerwa RD, Ellner JJ, Eisenach KD. 2000. Quantitative sputum bacillary load during rifampin-containing short course chemotherapy in human immunodeficiency virus-infected and non-infected adults with pulmonary tuberculosis. Int J Tuberc Lung Dis 4:528–536. 10864183

[37] Khan MS, Dar O, Sismanidis C, Shah K, Godfrey-Faussett P. 2007. Improvement of tuberculosis case detection and reduction of discrepancies between men and women by simple sputum-submission instructions: a pragmatic randomised controlled trial. Lancet 369:1955–1960. doi:10.1016/S0140-6736(07)60916-7 17560448

[38] Rieder HL, Lauritsen JM, Naranbat N, Katamba A, Laticevschi D, Mabaera B. 2009. Quantitative differences in sputum smear microscopy results for acid-fast bacilli by age and sex in four countries. Int J Tuberc Lung Dis 13:1393–1398. 19861012

[39] Chinnakali P, Selvaraj K, Thekkur P, Ramasamy G, Thulasingam M, Vasudevan K. 2014. Age and sex differences in sputum smear microscopy results for acid fast bacilli in a tertiary care centre, South India. J Respir Med 2014:1–5. doi:10.1155/2014/674942

[40] Burger ZC, Aung ST, Aung HT, Rodwell T, Seifert M. 2020. 658. Effect of HIV status on tuberculosis load as detected by Xpert MTB/RIF in sputum vs. saliva samples. Open Forum Infect Dis 7:S385–S386. doi:10.1093/ofid/ofaa439.851

[41] Muyoyeta M, Schaap JA, De Haas P, Mwanza W, Muvwimi MW, Godfrey-Faussett P, Ayles H. 2009. Comparison of four culture systems for Mycobacterium tuberculosis in the Zambian National Reference Laboratory. Int J Tuberc Lung Dis 13:460–465. 19335951

[42] Takeda M, Ito W, Kobayashi N, Konno K, Takahashi T, Tatsuko R, Tomita N, Tanigai T, Chiba T, Yamaguchi K, Sato K, Ueki S, Kayaba H, Chihara J. 2008. Co-existence of Mycobacterium tuberculosis and Mycobacterium intracellulare in one sputum sample. Intern Med 47:1057–1060. doi:10.2169/internalmedicine.47.0511 18520121

[43] Catanzaro A. 2002. Diagnosis, differentiating colonization, infection, and disease. Clin Chest Med 23:599–601. doi:10.1016/s0272-5231(02)00020-5 12370995

[44] Lumb R, Ardian M, Waramori G, Syahrial H, Tjitra E, Maguire GP, Anstey NM, Kelly PM. 2006. An alternative method for sputum storage and transport for Mycobacterium tuberculosis drug resistance surveys. Int J Tuberc Lung Dis 10:172–177. 16499256 PMC6522381

[45] Kolwijck E, Mitchell M, Venter A, Friedrich SO, Dawson R, Diacon AH. 2013. Short-term storage does not affect the quantitative yield of Mycobacterium tuberculosis in sputum in early-bactericidal-activity studies. J Clin Microbiol 51:1094–1098. doi:10.1128/JCM.02751-12 23345289 PMC3666816

[46] Paramasivan CN, Narayana AS, Prabhakar R, Rajagopal MS, Somasundaram PR, Tripathy SP. 1983. Effect of storage of sputum specimens at room temperature on smear and culture results. Tubercle 64:119–124. doi:10.1016/0041-3879(83)90036-3 6412408

[47] Kaboré A, Hien H, Sanou A, Zingué D, Daneau G, Ganamé Z, Nouctara M, Ouédraogo M, Ouédraogo O, Koutou F, Gomgnimbou M, Méda N, Neveu D, Godreuil S, Sangaré L. 2014. Impact of pre-analytical factors on mycobacterium cultures contaminations rates in Burkina Faso, West Africa. Pan Afr Med J 19:396. doi:10.11604/pamj.2014.19.396.5551 25995792 PMC4430149

