# Supplementary material for: Contamination rates in serially sampled sputum specimens obtained during tuberculosis treatment to capture culture conversion
Source: Microbiol Spectr. 2025 Aug 29;13(10):e00969-25. doi: 10.1128/spectrum.00969-25 (PMC12502799; doi:10.1128/spectrum.00969-25)
Supplement: Tables S1 and S2 — Stratified descriptive table and longitudinal model using the diagnostic laboratory definition. [file spectrum.00969-25-s0001.pdf]

Supplementary Tables:

Table S1. Average demographic, clinical, and collection condition characteristics stratified by sputum contamination status at week 1 (treatment initiation), using laboratory diagnostic definition. N=301

	median (IQR) or frequency (%)		
	Total n=301	Contaminated[a] n = 4	Non-contaminated n = 297
Male (sex)	180 (59.8)	2 (50.0%)	178 (59.9%)
Age (years, ref. <30)			
<30	87 (28.9)	0 (0.0)	87 (29.3)
30-39	77 (25.6)	1 (25.0)	76 (25.6)
40-49	73 (24.3)	1 (25.0)	72 (24.3)
>50	64 (21.3)	2 (50.0)	62 (21.3)
HIV positive (n=300)	85 (28.3)	2 (50.0)	83 (28.0)
Tobacco use	205 (68.1)	2 (50.0)	203 (68.4)
Smoked drug use[b]	165 (54.8)	0 (0.0)	165 (55.6)
Problem alcohol use[c]	186 (61.8)	2 (50.0)	184 (62.0)
Sputum volume (mL) (n=300)	2 (1,4)	1 (0,2)	2 (1,4)
Sputum color (yes) (n=300)	244 (81.3)	3 (75.0)	241 (81.4)
Supervised expectoration (n=276)	263 (95.3)	4 (100.0)	259 (95.2)
Time to culturing ≥2 days[d] (n=300)	142 (47.3)	3 (75.0)	139 (47.0)
Smear grade (ref. +++)[e]			
+++	85 (28.2)	0 (0.0)	85 (28.6)
++	48 (15.9)	0 (0.0)	48 (16.2)
+	48 (15.9)	0 (0.0)	48 (16.2)
Scanty	32 (10.6)	0 (0.0)	32 (10.8)
No AFB[f]	88 (29.2)	4 (100.0)	84 (28.3)
Culture Positive[g]	274 (91.0)	0 (0.0)	274 (92.3)
Cavitary TB disease[h] (n=294)	191 (63.7)	1 (25.0)	190 (64.2)

[a] Specimens considered contaminated only if a positive MGIT culture was observed to contain growth, but no *M. tuberculosis* on either ZN smear and/or blood agar (laboratory diagnostic definition)
[b] Defined as a positive urine drug test or self-reported use of methaqualone, methamphetamine, and/or cannabis
[c] Defined as a phosphatidylethanol (PEth) blood test >49 ug/L and/or an AUDIT score > 7
[d] Time from collection to processing and MGIT inoculation
[e] Observed with concentrated sputum smear microscopy
[f] Acid fast bacilli
[g] Presence of confirmed *M. tuberculosis*
[h] Lung cavitation identified using chest X-ray

Table S2: Longitudinal associations across the 12 treatment weeks. A logistic regression model was fitted to account for the within-participant correlation of contamination status, using laboratory diagnostic definition of contamination, from treatment initiation up to treatment week 12. N=2915

		OR[a]	95% CI[b]	p-value
Supervised expectoration		1.17	0.91, 1.53	0.214
Age (years, ref. <30)				
	30-39	0.84	0.59, 1.21	0.368
	40-49	0.80	0.56, 1.17	0.258
	> 50	0.97	0.66, 1.46	0.916
Smoked drug use		1.18	0.89, 1.59	0.239
Male sex		0.82	0.61, 1.12	0.220
HIV positive		1.49	1.13, 1.96	0.005
Treatment Week[c]		1.21	1.18, 1.25	<0.001

[a] Odds Ratio
[b] Confidence Interval
[c] Timepoint at which sample was collected by field or DOTS workers across a 12-week sampling period following treatment initiation
